# SL1 revisited: functional analysis of the structure and conformation of HIV-1 genome RNA

**DOI:** 10.1186/s12977-016-0310-9

**Published:** 2016-11-11

**Authors:** Sayuri Sakuragi, Masaru Yokoyama, Tatsuo Shioda, Hironori Sato, Jun-ichi Sakuragi

**Affiliations:** 1 Department of Viral Infections, RIMD, Osaka University, 3-1 Yamadaoka, Suita, Osaka 565-0871 Japan; 2 Pathogen Genomics Center, National Institute of Infectious Diseases, 4-7-1 Gakuen, Musashimurayama, Tokyo Japan

**Keywords:** HIV-1, RNA, Genome packaging, Dimerization, Recombination, Psi, Replication

## Abstract

**Background:**

The dimer initiation site/dimer linkage sequence (DIS/DLS) region of HIV is located on the 5′ end of the viral genome and suggested to form complex secondary/tertiary structures. Within this structure, stem-loop 1 (SL1) is believed to be most important and an essential key to dimerization, since the sequence and predicted secondary structure of SL1 are highly stable and conserved among various virus subtypes. In particular, a six-base palindromic sequence is always present at the hairpin loop of SL1 and the formation of kissing-loop structure at this position between the two strands of genomic RNA is suggested to trigger dimerization. Although the higher-order structure model of SL1 is well accepted and perhaps even undoubted lately, there could be stillroom for consideration to depict the functional SL1 structure while in vivo (in virion or cell).

**Results:**

In this study, we performed several analyses to identify the nucleotides and/or basepairing within SL1 which are necessary for HIV-1 genome dimerization, encapsidation, recombination and infectivity. We unexpectedly found that some nucleotides that are believed to contribute the formation of the stem do not impact dimerization or infectivity. On the other hand, we found that one G–C basepair involved in stem formation may serve as an alternative dimer interactive site. We also report on our further investigation of the roles of the palindromic sequences on viral replication. Collectively, we aim to assemble a more-comprehensive functional map of SL1 on the HIV-1 viral life cycle.

**Conclusion:**

We discovered several possibilities for a novel structure of SL1 in HIV-1 DLS. The newly proposed structure model suggested that the hairpin loop of SL1 appeared larger, and genome dimerization process might consist of more complicated mechanism than previously understood. Further investigations would be still required to fully understand the genome packaging and dimerization of HIV.

**Electronic supplementary material:**

The online version of this article (doi:10.1186/s12977-016-0310-9) contains supplementary material, which is available to authorized users.

## Background

The retrovirus genome is a single-stranded positive sense RNA. The viral genome always occurs as a dimer in virus particles, and the interaction is non-covalent, since heating easily dissociates purified dimeric genomes into monomers. It has been suggested that the encapsidation and dimerization of retroviral RNA genome plays an important role at various stages of the viral life cycle, including virion formation, reverse transcription and recombination.

Electron microscopy studies have suggested that the 5′ region of both viral RNAs contains a primary contact point and is referred to as the dimer linkage structures (DLS) [1–3]. The presumptive primary DLS of HIV-1 has been mapped to a region near the major splice donor and biochemical analysis indicates that the DLS probably consists of multiple stem-loop structures [4]. Within the DLS, stem-loop 1 (SL1) has been regarded as the most important region, which forms a stem-loop structure with a hairpin loop containing a six-nucleotide palindromic sequence [5]. The dimer formation would occur through a kissing hairpin mechanism by which the two RNAs would form an initial loop–loop contact based on complementary anti-parallel base pairing at the SL1 loops [6]. So far, several structural models of psi or DLS such as “seven stem-loops” [4], “branched multiple hairpin” [7] or “pseudoknot-like shape” [8] have been proposed. As SL1 is expected to be a highly stable structure, computer predictions have indicated the shapes of SL1 within the models are all rather similar. The reports performing chemical probing and mapping of viral RNA including SHAPE assay (selective 2′-hydroxyl acylation analyzed by primer extension) have proposed several total secondary structure models of HIV-1 psi [9–12]. Moreover, the standalone structure of SL1 and whole structure of HIV-1 psi have been solved and characterized by NMR to some extent [13–16]. However, there have been few attempts to pursue the precise functional structure of SL1 in the context of a live virus and thus the role of SL1 structure within the overall HIV-1 DLS in vivo is still not completely understood.

We previously developed a unique system to assess DLS operation within the HIV-1 virion and identified the region which is necessary and sufficient for HIV-1 genome dimerization within the virion [17]. In addition, we constructed a new packaging assay system based on the real-time RT-PCR with multiple probes. This system enabled a simple, reliable and reproducible quantification of HIV-1 genome-packaging efficiencies. Using these systems coupled with loss- and gain-of-function studies using site-directed mutagenesis, we performed the mapping of the functional base pairs within SL1 required for HIV-1 genome dimerization, packaging and replication, in much more further detail. Interestingly, the study revealed some unique information that has been never suggested previously. Based on these data, we made a modified SL1 structural model and also propose new possible processes of the HIV-1 RNA genome dimer formation. The importance of certain bases within SL1 is also discussed.

## Methods

### Constructs

The replication-competent HIV-1 proviral clone pNL4-3 (subtype B) [18], its *Env* mutant pNLNh [19], or its derivative DDNLp4∆2 (renamed as DDNd2) [17] were used as progenitors for the mutant constructs described below. Mutant plasmids were constructed with standard methods.

### DNA transfection

293T cells [20] (approximately 3 × 10^6^) were seeded on dishes (diameter, 100 mm) the day before transfection with plasmid DNA (5 µg) by means of the calcium phosphate precipitation method [21]. The day after transfection, the supernatant was replaced with fresh medium.

### Virus infection

At 48–72 h post-transfection, the media was centrifuged and supernatant was used for infection into MT-4 cells. Equal amounts of p24 were inoculated into the cells. For general experiments, 1 × 10^5^ MT-4 cells were infected with 20 ng-p24 amount of virus. For “low-moi” experiments, 1 × 10^5^ cells were infected with 4 ng-p24 amount of virus. The supernatants of the MT-4 were harvested every two to four days for multiple replication assays. Five microliters of each cell supernatant was subjected to exogenous RT assay as described previously [22].

### Isolation of RNA from virions and cells

At 48–72 h post-transfection, virus particles were collected from the media as described elsewhere [23]. The physical virus titer was determined with an ELISA assay kit for quantitation of CA-p24 (ZeptoMetrix, Inc., Buffalo, NY). To isolate RNA from particles, virions were disrupted by the addition of 1% sodium dodecyl sulfate (SDS) and treated with proteinase K (300 µg/ml) at room temperature for 60 min, followed by TE-saturated phenol/chloroform extraction, chloroform extraction, and ethanol precipitation.

### Real-time RT-PCR based packaging assay

To examine the accurate genome packaging efficiency of HIV, a real-time RT-PCR based packaging assay was developed. Basic concept is similar to an RNase protection assay with wild-type control [23]. To differentiate the control viral genome from those of the mutants of interest, a series of silent mutations for eight amino acids within CA region of pNLNh plasmid [24] were introduced and named as pNLNh-CAmut. This mutation did not affect virion production or genome packaging ability (data not shown.). A pair of primers to amplify the CA region (5′-ATCAAGCAGCCATGCAAATGTT-3′: FWdrosten and 5′-TGAAGGGTACTAGTAGTTCCTGCTATGTC-3′: RVdrosten) and two dual-labeled probes designed to anneal the CA region of either pNL4-3 or CAmut (5′CAL Fluor Red610-accatcaatgaggaagctgcagaatggga-3′BHQ-2: CAdrostenWT or 5′FAM-acTatTaaCgaAgaGgcAgcTgaGtggga-3′BHQ-1: CAdrostenMut, amino acid seq: TINEEAAEW) were synthesized (Biosearch Technologies, Inc. Petaluma, CA). A series of SL1 point mutants were each separately co-transfected into 293T cells along with pNLNh-CAmut as a control viral expression construct. Viral and cytoplasmic RNA isolated from the transfectant were applied for real-time RT-PCR with the primers and the probes. The PCR was performed with One Step PrimeScript RT-PCR kit and Thermal Cycler Dice (Takara Bio, Ohtsu, Japan). By comparing the amount of the control and the mutant RNA in virus and cell, it was possible to determine the effect of each of the mutations on relative encapsidation efficiency. Relative encapsidation efficiencies were calculated as the ratio of the amount of mutant to control (pNLNh-CAmut) genomic RNAs in the virions, with normalization to the cytoplasmic levels of the two RNAs.

### Northern blotting analysis

Pelleted RNA was resuspended in TE-buffer (10 mM Tris–HCl-pH7.5, 1 mM EDTA, 1% SDS, 100 mM NaCl, and 10% formamide) and the thermostability of dimeric viral RNA was determined by incubating RNA aliquots for 10 min at the prescribed temperatures [25]. RNA electrophoresis on native agarose gel and northern hybridization analysis were performed as described elsewhere [19]. Plasmid T7pol [25] was used to synthesize a complementary RNA probe for northern hybridization. In experiments designed to assess the conversion of dimers to monomers, relative amounts of both RNA species were quantitated by phosphorimager analysis (Fujifilm Co., Tokyo, Japan) to determine ratios of dimers and monomers. Since the ratio of monomer content to total RNA of virion varied from experiment to experiment, we calculated the “D value”, which is the index of dimerization efficiency, for validation. The calculation of D value was described elsewhere [19]. In short, we made formula (1) (below) to give an index value of dimer formation efficiency (D) for each of the mutants, to map the functional DLS area fairly and squarely.

1 $${\text{D}} = \left( {{\text{M}} - {\text{W}}} \right)/\left( {{\text{B}} - {\text{W}}} \right)$$

W represents ratios of monomer content to total RNA of virions at room temperature produced from DDNd2 (WT), which contains two wild-type DLSs on one viral genome. M represents that from each DDNd2 derivative mutant construct (generally carrying the mutation at the original position of DLS: 5′ side). B represents that from NLNh (Neg.) (Additional file 1: Figure S1). The D value of the Neg. is zero and that of WT is one in each experiment. When the combination of mutants severely affects dimer formation, the monomeric genome content of the virion is reduced and D becomes close to zero. Thus, the D value was expected to represent the magnitude of the effect of the DLS mutation in virions.

### Recombination assay

HIV-1 genome recombination assay was performed as described in our previous papers [26, 27]. In short, two similar vectors with the same or different dimerization signals (DLS) were constructed (Additional file 1: Figure S2A) and co-transfected together with or without the VSV-G expression vector (pCG-VSVG) (Additional file 1: Figure S2B). Vector A carries a surface biomarker (Mark A: murine HSA) and an inactivated eGFP gene with amino-terminal mutation (GFP∆N), while vector B carries another biomarker (Mark B: murine CD52) and an inactivated eGFP gene with carboxyl-terminal mutation (GFP∆C). After transfection, the released virions can be expected to co-package the homo- or hetero-dimerized vector genome, while the ratio of homo- to hetero-dimerization should be one-to-one if the genome expression efficiency of the two vectors is similar (Additional file 1: Fig. S2C). Without VSV-G, the expression of HSA and CD52 indicates transfection efficiency of the vectors, and no eGFP expression should be observed. With VSV-G expression, pseudotyped virions have been observed to cause retro-transduction to the producer cells [28] and the number of marker genes expressing cells in the transfectant increases with an increase in the occurrence of retro-transduction. Recombination of the two vectors can be assumed to occur only in retro-transduced cells, and is monitored in terms of further restoration and expression of the eGFP gene (Additional file 1: Figure S2C). Transfection and retro-transduction efficiency are measured by expression of Mark-A and -B, while the transduction efficiency is estimated by subtracting marker gene expression ratios of the VSV-G negative sample (bA, bB%) from those of the positive sample (rA, rB%). Finally, the recombination efficiency is estimated by calculating the ratio of eGFP expressing cells (rG–bG%) in vector-transduced cells. If we assume that one of the recombination events always occurs between the ∆N and ∆C mutation of eGFPs during reverse transcription, 50% of the recombination events should result in reconstitution of the eGFP gene. In addition, 50% of the virions from doubly transfected cells possess a hetero-dimerized genome, and thus have the potential to reconstitute eGFP. This means that a ratio of eGFP positive cells of 25% in Mark-A or -B positive cells should be the maximum value for recombination. We therefore adopted this maximum ratio for easy indexing by quadrupling the numeric results (Additional file 1: Figure S2C).

### Construction of three-dimensional (3D) structural model of the SL1

Computer-aided 3D models were constructed by the software MOE(Molecular Operating Environment: Chemical Computing Group Inc. Montreal, Canada) using portions of reported monomer and dimer structures of the DLS region to incorporate information regarding nucleotide base pairs in Fig. 7.

- monomer: pdb id = 1N8X [14]
- dimer: pdb id = 2B8R (X-ray, resolution 2.6A [29])

## Results

**Fig. 1:**
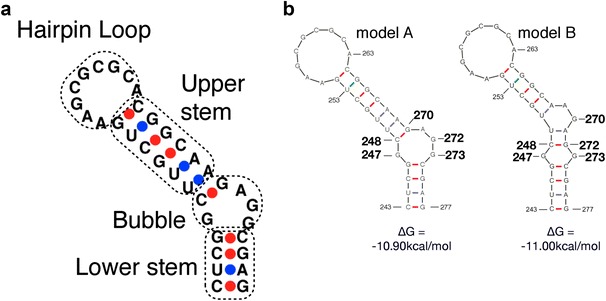
Secondary structural models of SL1. **a** Dissection of SL1 applied for this report. **b** Two models of SL1 predicted by Mfold program. The nucleotides at the bubble region substituted in the mutants are numbered. Five single substitution mutants and three double substitution mutants were constructed

Based on the structural models proposed by computer predictions and previous studies [14, 30–32], we dissected SL1 into four parts, a top loop composed by nine nucleotides, upper and lower stems, and several free bases between the two stems (Fig. 1a). We named them as “hairpin loop”, “upper stem”, “lower stem”, and “bubble” respectively, and attempted to examine the importance of each base or basepairing for viral activity by introducing base substitution mutations. We measured viral RNA dimerization efficiency and RNA packaging efficiency by our previously-reported assay protocol [17, 19], while also performed viral replication assay using MT4 cells to measure infection. RNA recombination assays [26] were also applied for some mutants to elucidate more precise detail.

### Determination of the “bubble” shape and its contributing nucleotide bases: Which is the actual in vivo form?

**Fig. 2:**
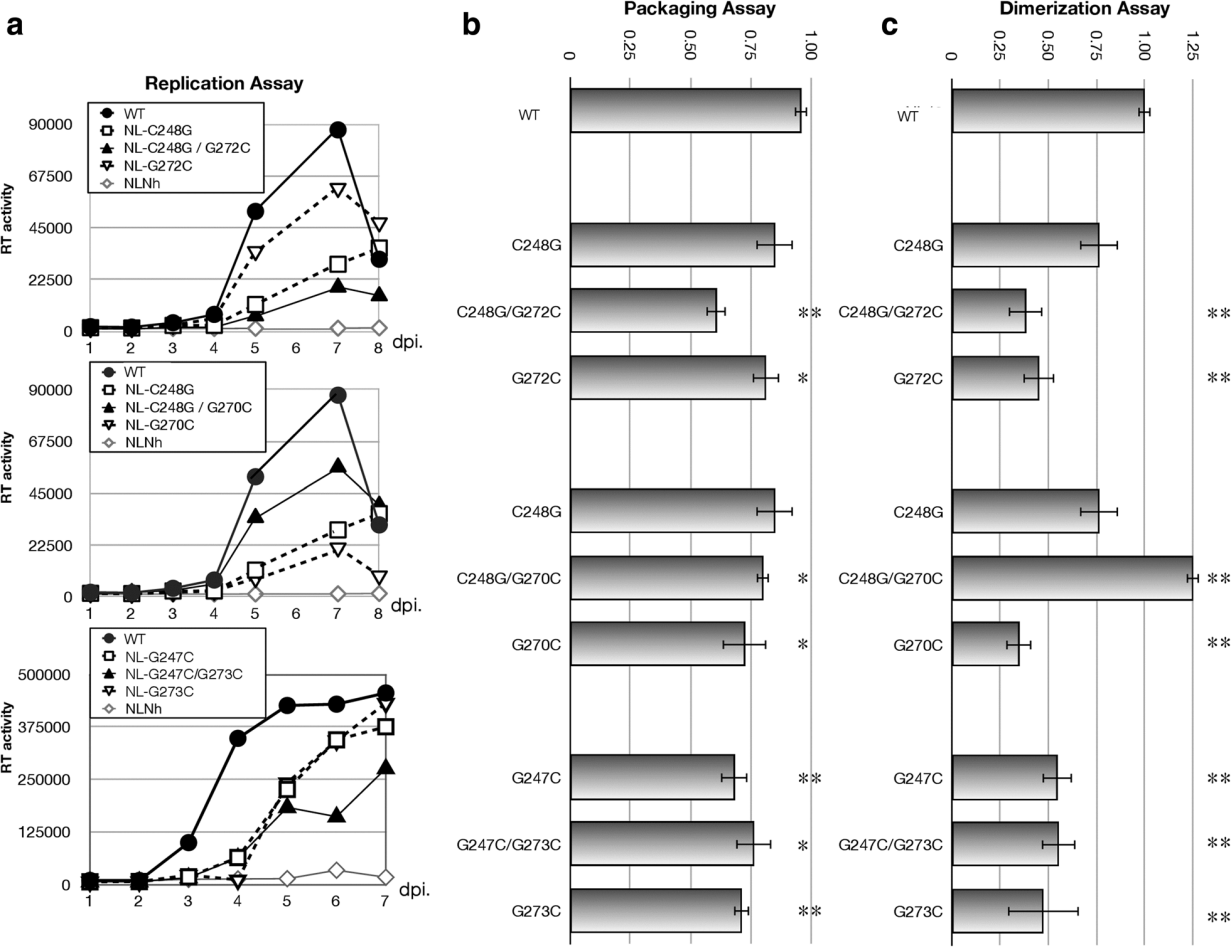
Confirmation of the computer prediction model. **a** Growth kinetics of the mutants. At least three independent experiments were performed and similar results were obtained. One of the representative data for each mutant was shown. The molecular infectious clone, NL43 (the wild-type: WT) and its env mutant, NLNh, was put as positive and negative controls in each experiment respectively. The X-axis represented the days post infection (dpi), and the Y-axis represented the RT activity (PSL) in cell supernatant measured by Imaging Plate (Fujifilm, Japan). **b** Packaging efficiency of each mutant. The Y-axis represented the magnitude of efficiency of each mutant compared to that of the control vector, NLNh-CAmut (see “Methods” section). **c** Dimerization ability of each mutant. The Y-axis represented the magnitude of efficiency of each mutant compared to that of the wild-type (WT). The value of the WT was set as one. At least three independent experiments were performed. The data shown were the average of the value and error bars represented SEM. *Statistical significance compared to the wild-type (WT) at *p* < 0.05, and **statistical significance at *p* < 0.01

Computer programs often predict multiple RNA secondary structures within a specific RNA region. In the case of SL1 region, there are two major models, A and B (Fig. 1b). Model A is a well-accepted structure and acts as the actual consensus structure model thus far. On the other hand, the enthalpy (∆G) of model B is slightly lower than that of model A and the Mfold program [33] has a preference to form model B. As there is little information about the base pairings around the Bubble region that contribute to viral activity, including RNA dimerization and packaging, we constructed several base substitution mutants and examined the role of these bases (Fig. 1b). A mutation G272C disrupts C248–G272 basepairing existing in model B and C248G/G272C reconstitutes it. Similarly, the mutants G270C and C248G/G270C were constructed to examine the validity of model A. We also introduced substitution mutations in G247 or G273 to cytidine to determine their functional roles. As shown in Fig. 2a–c, viral replication, RNA dimerization and packaging levels of G272C and C248G were reduced to a certain degree and the double mutant C248G/G272C showed additional defects especially in packaging and replication functions. Meanwhile, the replication and dimerization functions of C248G/G270C were efficiently restored compared to those of the single mutants (C248G and G270C). Any mutants that have mutation(s) in G247 or G273 lacked wild-type level function. These data suggested that model A appeared to be a functional structure in vivo, although its stability is slightly lower than that of model B. The guanines in the bubble (G247, G272 and G273) play very important roles on both genome dimerization and virus replication, but their contributions to SL1 structure yet remain unclear.

### Contribution of G–C pairs in the upper stem

**Fig. 3:**
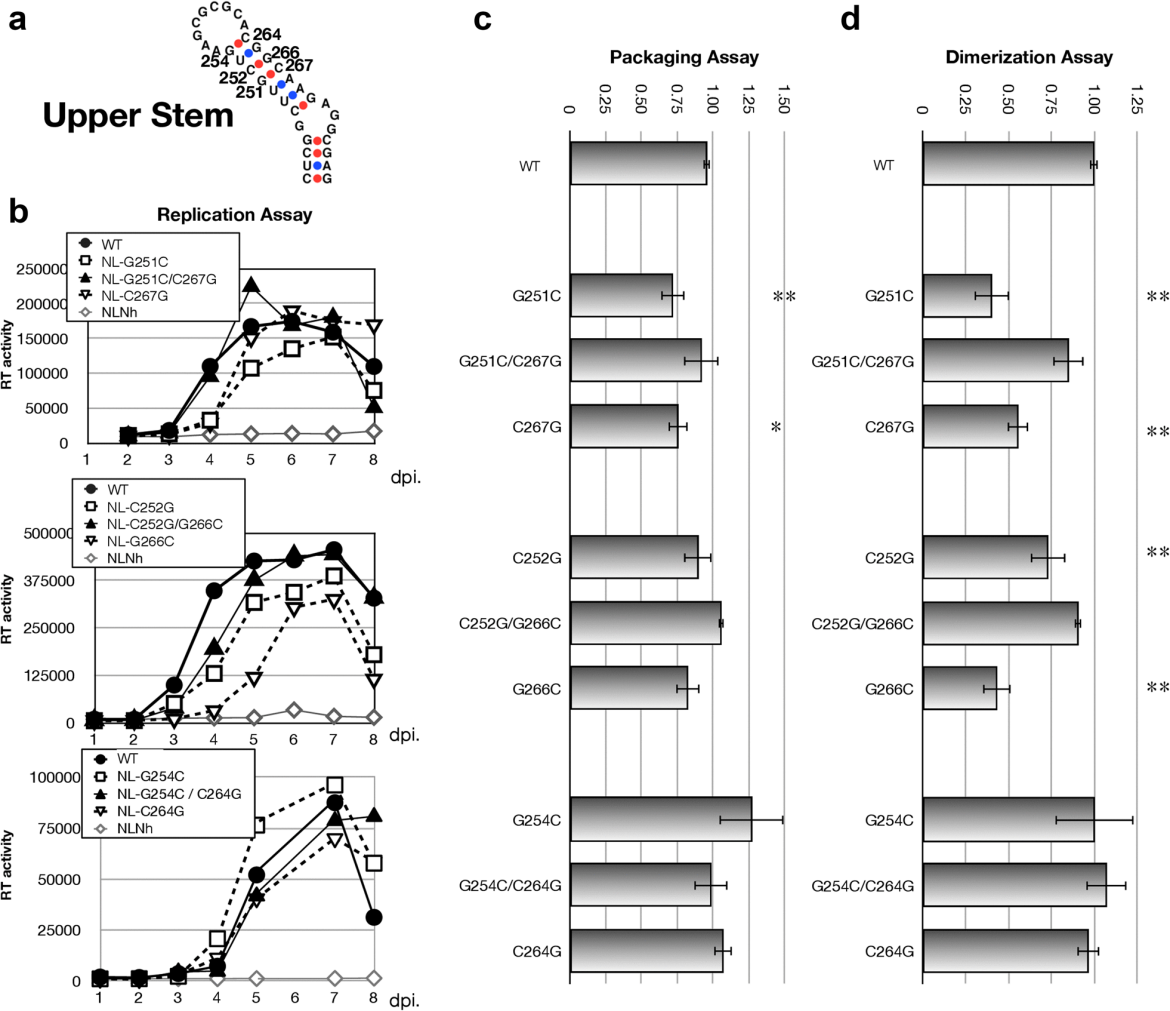
Inspection of G–C base pairing in the upper stem region of SL1. **a** The nucleotides substituted in the mutants. Six single substitution mutants (G to C or C to G) and three double substitution mutants (G–C to C–G or C–G to G–C) were constructed. **b** Growth kinetics of the mutants. **c**, **d** Packaging efficiency and dimerization ability of each mutant. The experiments were similarly performed as described in Fig. 1

We next examined functional contribution of three G–C basepairings at the upper stem (Fig. 3). We constructed two single substitution mutants to disrupt basepairing and one double mutant to restore hydrogen bonding for each basepair (Fig. 3a), and then examined the contributions of each base to viral activity (Fig. 3b–d). The lower two basepairings, G251–C267 and C252–G266, showed similar results. Both double mutants restored all of their activity to wild-type levels whereas most of single mutants indicated reduced viral activity. Unexpected results were obtained from the mutants G254–C264. None of the mutations at this position affected viral replication and other functions. These data might suggest that it is not essential to form a basepair of this position for viral replication.

Overall in the upper stem, G251–C267 and C252–G266 basepairings appeared to occur in vivo, while on the other hand, the basepairing of G254–C264 might be dispensable for viral activity.

### Contribution of G–C pairs in lower stem

**Fig. 4:**
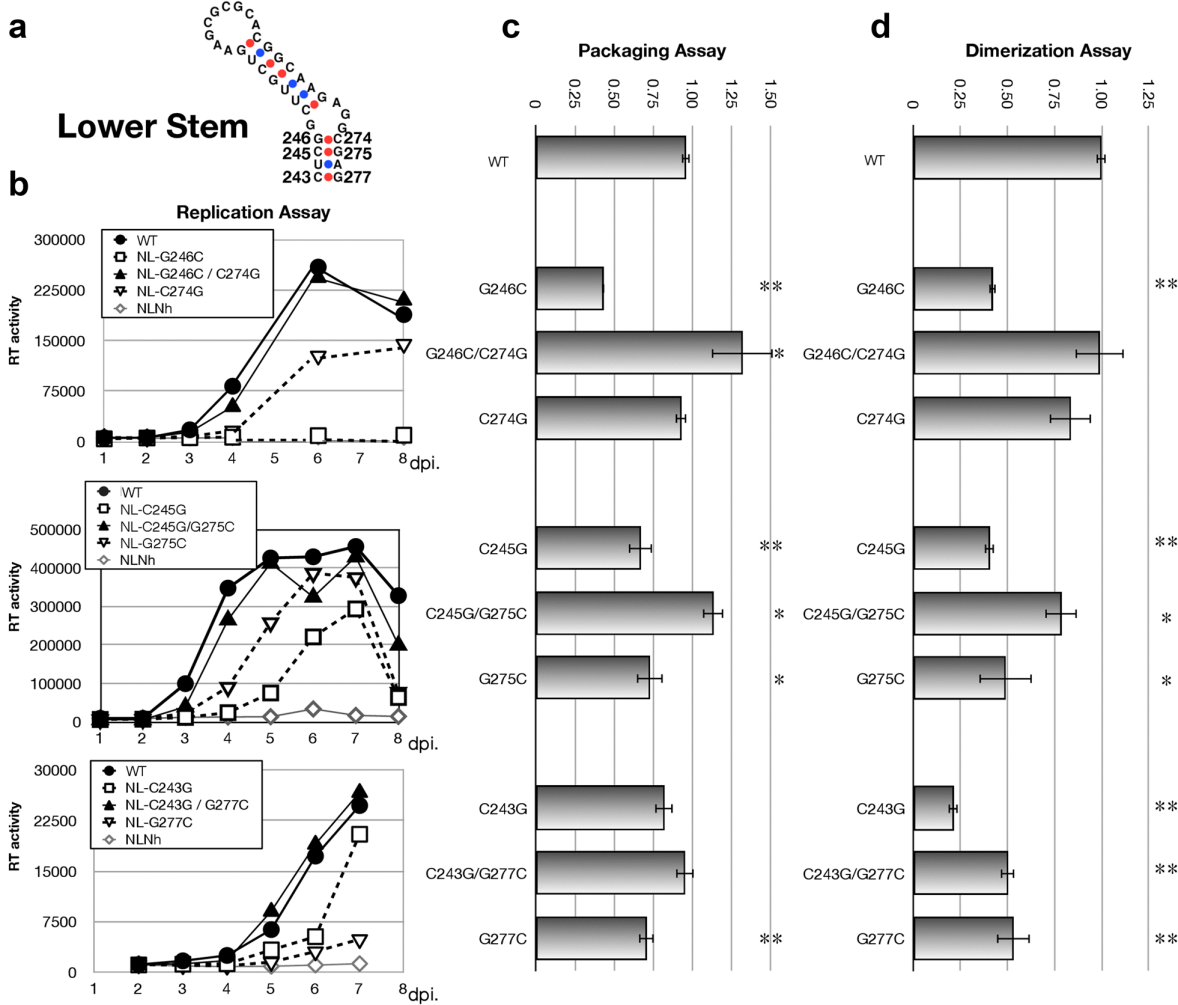
Inspection of G–C base pairing in the lower stem region of SL1. **a** The nucleotides substituted in the mutants. Six single substitution mutants (G to C or C to G) and three double substitution mutants (G–C to C–G or C–G to G–C) were constructed. **b** Growth kinetics of the mutants. **c**, **d** Packaging efficiency and dimerization ability of each mutant. The experiments were similarly performed as described in Figs. 1, 2

**Fig. 5:**
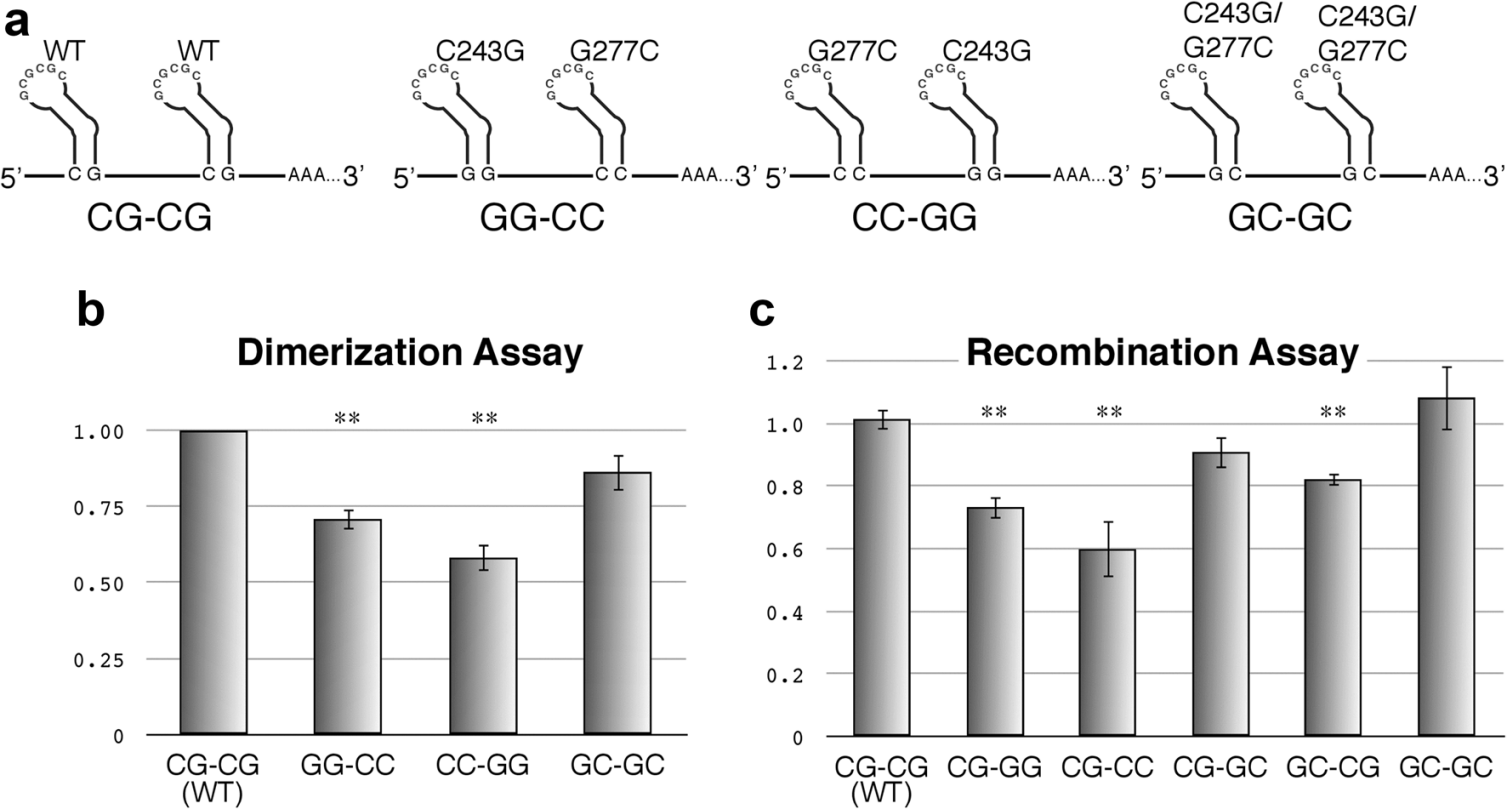
Investigation of the role of C243–G277 base pair on dimer formation. **a** Schematic representation of the mutants constructed for dimerization assay. **b** Dimerization ability of each mutant. The Y-axis represented the magnitude of efficiency of each mutant compared to that of the wild-type. The value of the wild-type was set as one. **c** Genome recombination assay with various combinations of the mutants. The Y-axis represented the magnitude of recombination in each assay. The value of the homomeric recombination of wild-type (CG–CG) was set as one

We next examined the functional contribution of three G–C basepairings within the lower stem (Fig. 4). We constructed two single-substitution mutants to disrupt basepairing and one double mutant to restore hydrogen bonding for each basepair (Fig. 4a), and then examined the contributions of each base to viral activity. The two basepairings, G246–C274 and C245–G275, showed similar result. Both double mutants restored most of their activities to wild-type levels, whereas the single mutants indicated reduced viral activity. On the other hand, a noteworthy discrepancy was found in the C243–G277 basepairing, located at the bottom of the stem. The C243G/G277C double mutation did not fully restore wild-type levels of dimerization (Fig. 4d), but viral replication and packaging efficiency were restored (Fig. 4b, c). A possible explanation was hypothesized to explain this paradox. The dimerization efficiency assay system is based on measuring the interaction between two DLS, where one is a mutant and the other is the wild-type (Additional file 1: Figure S1) [8, 17], enabling us to discover the mutation(s) which disrupt “the functional DLS structure”. However, if the mutation did not affect the DLS functional structure formation, but the DLS lost its ability to interact with wild-type DLS, the system would give us similar results as the aforementioned DLS disruption. To verify this explanation, we constructed several mutants that measure the interaction between two mutant DLSs (Fig. 5a). As expected, homo dimerization between the C243G/G277C double mutant DLSs showed a fairly efficient interaction comparable to that of the wild-type (Fig. 5b). We further examined the effect of C243G/G277C mutation on genome recombination utilizing our assay system [26] since genome dimerization and recombination are suggested to be tightly linked [27]. Various combinations of the mutants carrying C243–G277 were examined for their ability of hetero/homo genome recombination during infection (Fig. 5c). The homo-genome combination between wild-types or between C243G/G277C mutants showed the full recombination rate, suggesting that the C243–G277 basepair plays a important role in the intermolecular interactions between the two DLSs.

### Effect of hairpin loop modification to disrupt kissing-loop interaction

**Fig. 6:**
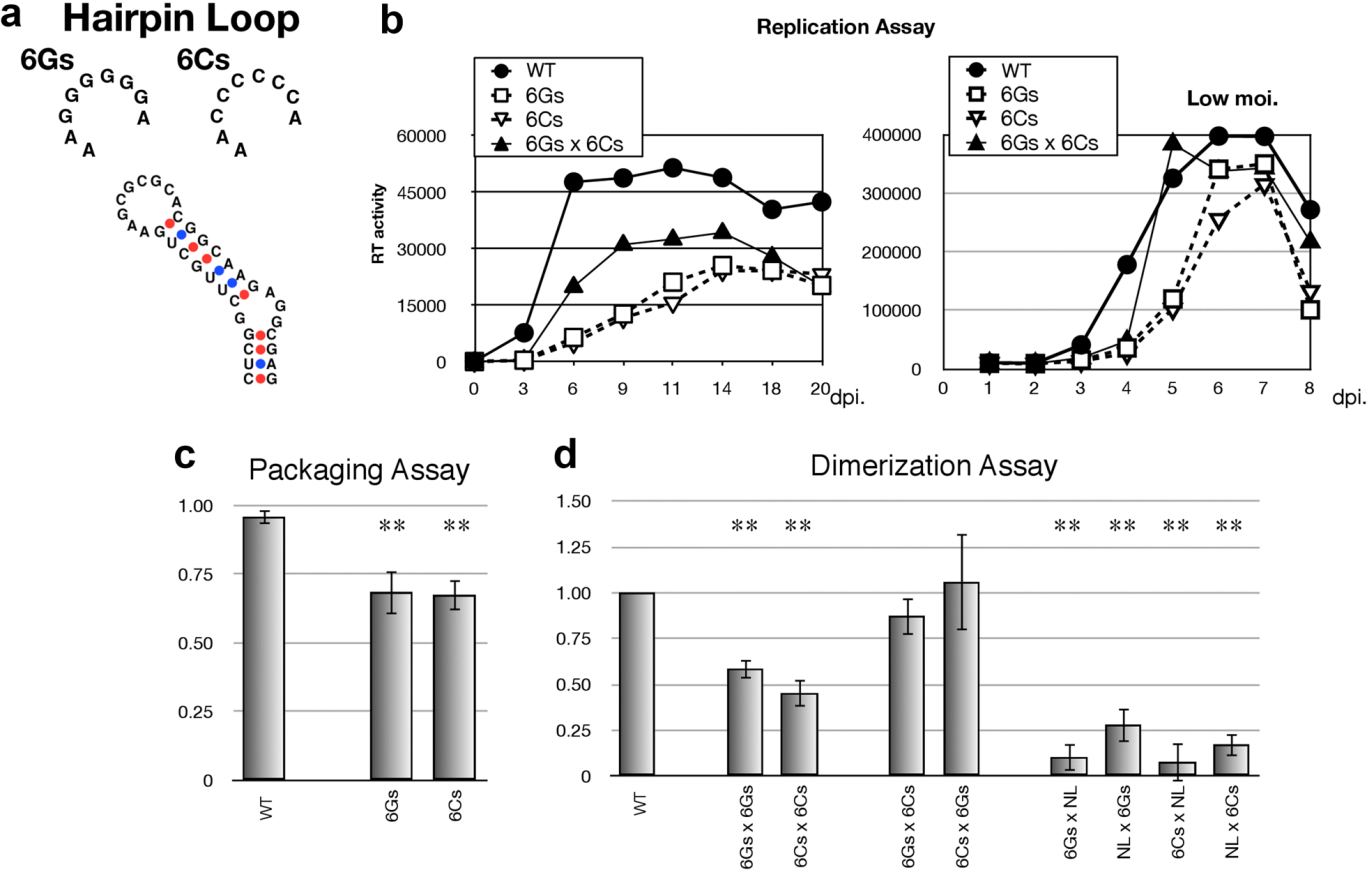
Inspection of the functional roles of six-base palindrome at the hairpin loop region of SL1. **a** The nucleotides substituted in the mutants. Six-base palindrome (GCGCGC) was substituted by 6Gs (GGGGGG) or 6Cs (CCCCCC). **b** Growth kinetics of the mutants. In addition to the wild type and two mutants, a virus obtaining hetero-dimerized genome (6Gs × 6Cs) were produced by co-transfection with two plasmids and applied for infection of MT-4 cells. Representative viral growth curve with standard (*left*) and low moi (*right*) experiments were shown. **c**, **d** Packaging efficiency and dimerization ability of each mutant. The experiments were similarly performed as described in Figs. 1, 2

The six-nucleotide palindromic sequence at SL1 hairpin loop is believed to play a central role for genome dimerization and packaging by mediating the initial physical contact of two RNA strands [6]. We changed GCGCGC sequence of the wild-type to GGGGGG (6Gs) or CCCCCC (6Cs) (Fig. 6a) and further validated its importance to viral activity. We first examined the replicative abilities of these mutants and the effects of co-packaging these mutated genomes. The replicative abilities of these mutants were crippled and the virus produced from the cells co-transfected with 6Gs and 6Cs showed restoration of replication regardless of moi condition (Fig. 6b). Similarly, the genome packaging and dimerization efficiency of the mutants were reduced, and only the combination of six base pairing of kissing loop (6Gs × 6Cs) rescued genome dimerization comparable to wild-type levels (Fig. 6c, d). These data suggested that co-existence of discordant or accordant hairpin loops of psi predictably affected viral activity and complete base pairing of the hairpin loop is required for efficient viral replication, which are consistent with the current consensus.

## Discussion

There are many reports concerning HIV-1 psi RNA structure employing various technologies nowadays [9–16]. Although such technologies have given us notably information, they have limitations for complete realization of the structure within virion or cell. For example, the primary information provided by RNA mapping with chemical probing is only that which nucleotides form double strand or not. Solving molecular structure by crystallography or NMR is performed in in vitro condition, which might not fully reflect them in native status. Thus even simple and ordinary methods, precise mutagenesis of psi RNA and analysis on viral functions of the mutants are still significant actions for understanding an overall structure of psi RNA in virion. In this report, we successfully analyzed precise roles of G–C pairings within the HIV-1 SL1 region and found several possibilities of novel genomic structures, which may have functional merits.

In the bubble region, we confirmed that the base pairing of C248–G270 was very important for viral activity and for model A (Fig. 1b) which appeared to be the functional structure of SL1. Contrary to the suggestions from computer predictions, SL1 forms the bubble with 3–1 bases (Fig. 1b: model A) in vivo, despite showing slightly lower stability. We were also interested in the non-basepairing guanines at the bubble. We constructed and examined two mutants, which were forced to form a base-pair (G247C and G273C) and a mutant which possesses cytidines instead of guanines (G247C/G273C) at the bubble. As a result, none of the mutants retained full viral activity, suggesting that the exposed guanines at the bubble are important for both dimerization and viral replication. As several reports have implicated [10, 34–38], the preference manifested by NC for unpaired guanine bases may contribute to the RNA-NC interaction at the beginning of dimerization and the formation of the model A structure.

Upon investigating the upper and lower stems, most of the G–C pairings in SL1 stem appeared to be important for SL1 function as expected (Figs. 3, 4). Surprisingly, one pairing proximal to the hairpin loop (G254–C264) seemed to be tolerable for base substitution or dispensable (Fig. 3). As this G–C interaction limits the size of the loop, this data might suggest that the putative loop is larger than initially believed, composed by eleven exposed nucleotides. Since the six-nucleotide palindrome must be aligned antiparallelly for the initiation of dimer formation, the flexibility of the hairpin loop attributed by these non-pairing bases should yield some advantages to achieve kissing-loop formation.

Additional unexpected results were obtained from one basepair at the bottom of the lower stem (C243–G277). The exchange of these bases (C243G–G277C) did not affect viral replication and genome packaging, but the ability of the mutated DLS to interact with wild-type DLS was notably reduced (Fig. 4). The following experiment revealed that upon concordance of intermolecular basepairing (GG–CC, CC–GG, or GC–GC) of this position showed some restoration of dimerization efficiency compared to those of the discordant pairs (Figs. 4d, 5b). Especially, homo-dimerization of the double mutant DLS (GC–GC) was sufficiently comparable to that of the wild-type. In addition, the genome recombination rate between homomeric double mutants was also similar to that of the wild type (Fig. 5c). From these data, we consider that the bottom portion of SL1 might operate at an alternative dimer interface—at least transiently—during some steps of genome dimerization. There was a discrepancy also between replication and dimerization of the mutants. The mutant C243G showed much less dimerization ability compared to that of G277C whereas viral replication of C243G was better than that of G277C (Fig. 4b, d). We think there are two possibilities. One is that the packaging efficiency (C243G > G277C) is more important than the dimerization ability (C243G < G277C) for replication of the mutants in this case. The other is, which is most likely, that the results of dimerization efficiency of the mutants concerning C243–G277 in Fig. 4d might not reflect the actual dimerization ability of them in virion. As explained in the “Results” section, our dimerization assay system measures the hetero-dimerization between a mutant and the wild-type DLS (Fig. 5a, Additional file 1: Figure S1C). As described in Fig. 5b, homo-dimerization ability of C243G/G277C mutant is far different from the result in Fig. 4d. Thus, although we do not have the data, it is possible that homo-dimerization efficiency of C243G single mutant genome in virion is not as low as the data shown in Fig. 4d.

In the upper or lower stem, all mutants having exchanged base pairs (G–C to C–G or vise versa) did not alter the viral activity much. This suggests that base pair formation is important for SL1 function, but the specific base location is not, with the exception of the bubble guanines.

Complete base pairing between two hairpin loops of SL1 appeared to be important for efficient viral replication (Fig. 6). Co-existence of 6Gs and 6Cs genome in virion restored viral replication and dimerization (Fig. 6b, c). These data suggested that the virion preferably packages hetero-dimerized genome with complete basepairing in this case and thus efficient genome packaging helped viral replication. Although it is consistent with the currently-accepted theory [39], evidence of some replicative ability was observed in 6Gs and 6Cs mutants, albeit it was reduced and delayed. As the necessity of DIS loop for virus replication was previously discussed in some reports [40, 41], this might suggest that kissing loop interaction is not absolutely required for virus replication or that the kissing loop is somehow formed even between non-complimentary strands to establish a functional psi in a certain condition.

**Fig. 7:**
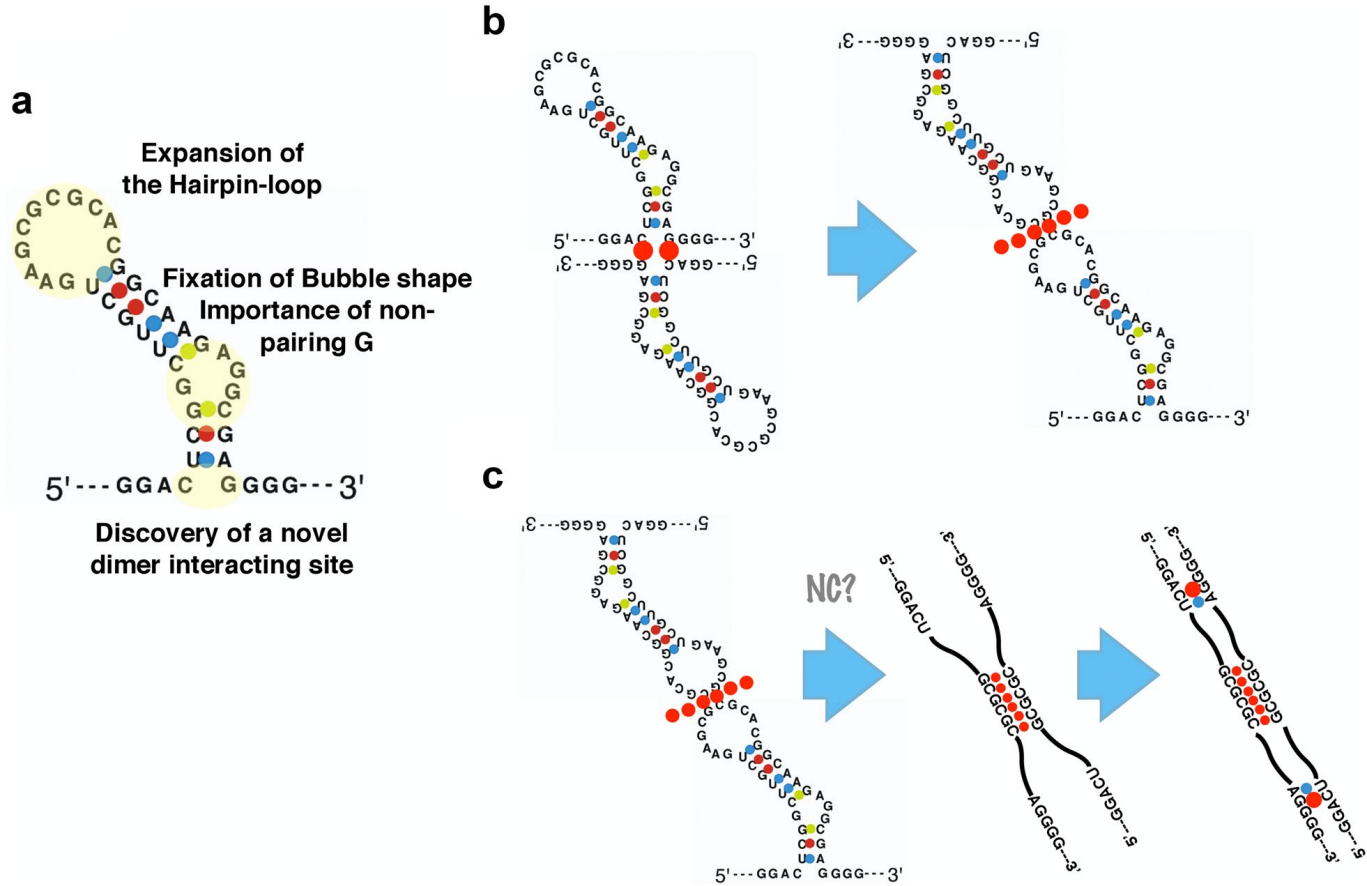
Schema of SL1 roles on genome dimerization. **a** Modified structural model of SL1 based on the results. **b**, **c** Hypothesis of G243/C277 function. **b** The bases interact intermolecularly before DISs make kissing dimer. **c** The bases form base pairs to knot and stabilize an extended duplex of SL1

Collectively considering all the previous data, we suggest a modified SL1 secondary structural model (Fig. 7a). Although the majority of the shape is similar to model A (Fig. 1b), the hairpin loop is two bases larger and the bottom of the stem (G243/C277) works to interact with the other DLS molecule. We named this possible interface as “dimer interactive site” (DIntS) and considered how DIntS works to establish dimer during virion formation. It is impractical to presume that DIS–DIS and DIntS–DIntS interactions occur at the same time within DLS dimer since two DIntS should be located at opposite side each other in the kissing-loop structure model [29]. Thus we suggest several possibilities, as to when the DIntS interaction occurs. The first one is the “pre-initiation model”, where DIntS mediates the primary interaction of DLS dimer formation and after the two molecules are sufficiently proximal, DIS interaction occurs (Fig. 7b). However, in the context of the full-length RNA these regions are paired and forming rather complicated structures [16], hence it seems severe that interaction can happen. Another possibility is the “finalization model”. After interaction with DIS, two DLSs start to “melt” together by the viral nucleocapsid protein to form an extended duplex [42–44]. As DIntS positions are at the very end of the extended duplex of SL1, intermolecular basepairing of DIntS would play a critical role to make the extended duplex tight and stable. Recent report suggested the implication of the upstream flanking nucleotides of SL1 to extended duplex formation [45] and might support our hypothesis. Third idea is the “structure-maintenance model”. According to the SHAPE data from Wilkinson et al. [10] bases 239–242 and 278–279 are unpaired and form a bulge (the lower bulge) that is stabilized by 236–239 and 280–282 (the ground stem). This particular structure is very pertinent to the results presented in our results. Changes in C243 or G277 could destabilize the ground stem and/or bend the structure of the lower bulge, this having very strong effects on the overall stability of SL1. In addition, although G–C pairing at 243–277 is restored in double mutant (C243G/G277C), the whole structure of SL1 including the ground stem and the lower bulge could be modified not to interact with the wild-type SL1 but only be able to match with same mutant each other. This hypothesis could also explain our data concerning homo-and hetero-dimerization of 243–277 mutants.

**Fig. 8:**
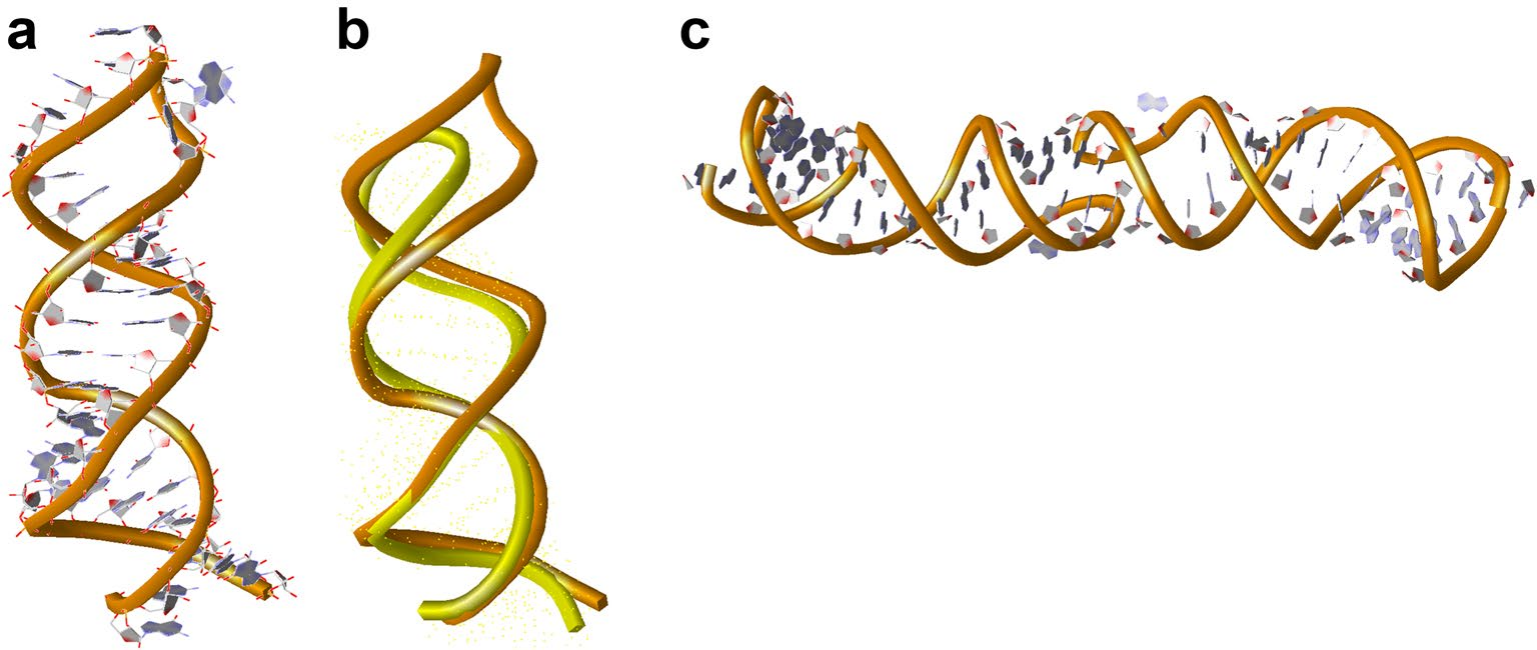
Computer-aided 3D models of SL1. **a** Model constructed using parts of reported monomer structure of SL1 (pdb id = 1N8X) to incorporate the information about nucleotide base pairs shown in Fig. 7a. **b** Superimposition of the Fig. 7a structure with the authentic SL1 structure (pdb id = 1N8X). **c** The modified dimer model using the Fig. 7a structure and parts of reported dimer structure (pdb id = 2B8R). The representations of A and C include nucleotide side chains

Based on Fig. 7, computer-aided RNA molecule models were constructed (Fig. 8) and molecular dynamics (MD) simulation pertaining to the stability of the models were performed. Figure 8a is a 3D model of Fig. 7a and it was superimposed with the traditional SL1 model (Fig. 8b) [14]. Figure 8c is 3D model of the schema of Fig. 7c right. As seen in Fig. 8b, the height of the proposed model is greater than the traditional model. This might be favorable for dimer initiation since SL1 must protrude from the DLS region to increase the likelihood of contacting its partner. After MD simulation, all models shifted their shapes to the traditional model and restored C243–G277 and G254–C264 bonds, even though model 7C maintained a dimer form. These results suggested that the formulation of the proposed SL1 models require some assistance by RNA-binding cofactors, which may indicate a role for Gag precursors.

## Conclusion

We performed a series of precise, functional mapping studies of SL1 in HIV-1 DLS and discovered several possibilities for a novel structure. In the newly proposed structure, the hairpin loop of SL1 appeared larger, and DIntS may mediate a more complicated mechanism of genome dimerization than previously understood. A recent report suggested that HIV-1 Gag precursor Pr55 preferentially binds to the free guanines at the bubble and at the lower stem of SL1 [46]. These data are consistent with our own results and also imply the possibility of important roles of the lower stem of SL1 on intermolecular interactions. Further investigations would be still required to fully understand the genome packaging and dimerization of HIV.

## Additional file

**Additional file 1: Figure S1.** Native northern blots of HIV-1 genome RNA in virion.** Figure S2.** The schema of the system for estimating HIV-1 recombination efficiency.
