# Supplementary figures and images for: SL1 revisited: functional analysis of the structure and conformation of HIV-1 genome RNA
Source: Retrovirology. 2016 Nov 11;13:79. doi: 10.1186/s12977-016-0310-9 (PMC5106843; doi:10.1186/s12977-016-0310-9)

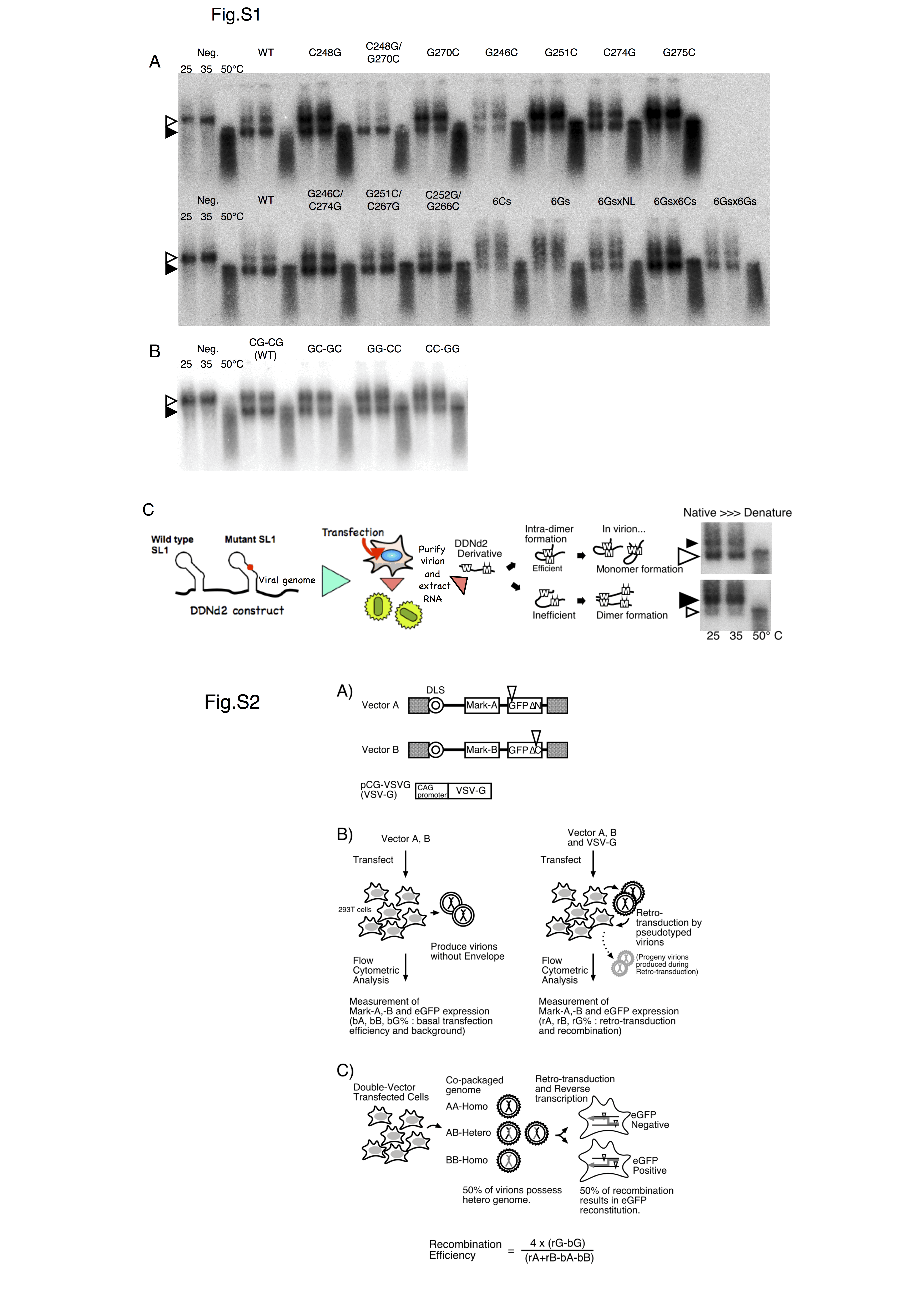

Supplement: Supplementary file 1 — Additional file 1: Figure S1. Native northern blots of HIV-1 genome RNA in virion. Figure S2. The schema of the system for estimating HIV-1 recombination efficiency. [file 12977_2016_310_MOESM1_ESM.gif]
